# Editorials

**Published:** 1893-08

**Authors:** 


### HOMŒOPATHIC PHYSICIAN,

A MONTHLY JOURNAL OF

HOMŒOPATHIC MATERIA MEDICA AND CLINICAL MEDICINE.

"If our school ever gives up the strict inductive method of Hahnemann, we are lost, and deserve only to be mentioned as a caricature in the history of medicine."—CONSTANTINE HERING.

Vol. XIII. AUGUST, 1893. No. 8.

### EDITORIALS.

PYROGEN. is one of the nosodes. It has not been very cordially received by the profession hitherto, not alone because of its revolting character, but because it has been insufficiently proved. The introduction of this nosode is due to Dr. Samuel Swan, of New York. Nearly all that has been known about it hitherto is due to his labors. In the April number of this journal, at page 204, Dr. Sherbino has given some valuable clinical symptoms. In the present issue of THE HOMŒOPATHIC PHYSICIAN will be found an excellent article by Dr. W. A. Yingling, in which are collected in order all the reliable symptoms obtainable. The authority for most of these symptoms is also given.

The profession will now be able to prescribe this valuable remedy intelligently. A glance over these symptoms will recall to the practitioner many a case where symptoms cropped out that he could not assign to any remedy, and for want of this knowledge his case has made a tardy recovery. He will now know the indicated remedy when he meets these symptoms again.

---

HOMŒOPATHIC AGGRAVATION is the title of an excellent article by Dr. S. E. Chapman, which appears in this number. It is cordially commended to our readers for careful perusal. Dr. Chapman forcibly illustrates what has been before said in these editorials that the physician who fails to carefully select the remedy by a close comparison of the symptoms, who fails, in short, to study the materia medica and apply it, for him the genius of Homœopathy is a sealed book. Unconscious of his own shortcomings in this regard and confronted, on the other hand, with unsolved clinical problems in the persons of suffering patients, he resorts to methods of treatment borrowed from old-school rational therapeutics, and so finds Homœopathy ineffectual.

On the other hand, he who carefully learns the tenets of *The Organon*, and as carefully fits his materia medica to the case in hand, is "born into the kingdom," as Dr. Chapman expresses it, and derives a satisfaction, and is permeated with a deep conviction of the truth of the principle he practices, to which every other practitioner is a stranger. Here then is the gulf that separates the two orders of mind and renders their differences irreconcilable.

